# Brief report: Effect of cardiac multi-morbidity on COVID hospitalization outcomes

**DOI:** 10.1371/journal.pone.0301898

**Published:** 2024-04-24

**Authors:** Fouad Chouairi, Edward Jaffe, Abdul Mannan Khan Minhas, Marat Fudim

**Affiliations:** 1 Department of Medicine, Duke University School of Medicine, Durham, NC, United States of America; 2 George Washington University Hospital, Washington, District of Columbia, United States of America; 3 Division of Medicine, University of Mississippi Medical Center, Jackson, MS, United States of America; 4 Division of Cardiology, Department of Internal Medicine, Duke University School of Medicine, Durham, NC, United States of America; 5 Duke Clinical Research Institute, Durham, NC, United States of America; Children's National Hospital, UNITED STATES

## Abstract

**Background:**

The COVID-19 pandemic has stretched healthcare resources thin and led to significant morbidity and mortality. There have been no studies utilizing national data to investigate the role of cardiac risk factors on outcomes of COVID hospitalizations. The aim of this study was to examine the effect of cardiac multimorbidity on healthcare utilization and outcomes among COVID hospitalizations during the first year of the pandemic.

**Methods:**

Using the national inpatient sample (NIS), we identified all adult hospital admissions with a primary diagnosis of COVID in 2020, using International Classification of Diseases, Tenth Revision, Clinical Modification codes (ICD010-CM). Coronary artery disease, diabetes mellitus, heart failure, peripheral vascular disease, previous stroke, and atrial fibrillation were then identified as cardiac comorbidities using ICD-10-CM codes. Multivariable logistic regression was used to evaluate the effect of cardiac multimorbidity on mortality and mechanical ventilation.

**Results:**

We identified 1,005,040 primary COVID admissions in 2020. Of these admissions, 216,545 (20.6%) had CAD, 413,195 (39.4%) had DM, 176,780 (16.8%) had HF, 159,700 (15.2%) had AF, 30735 (2.9%) had PVD, and 25,155 (2.4%) had a previous stroke. When stratified by number of comorbidities, 428390 (40.8%) had 0 comorbidities, 354960 (33.8%) had 1, 161225 (15.4%) had 2, and 105465 (10.0%) had 3+ comorbidities. COVID hospitalizations with higher cardiac multimorbidity had higher mortality rates (p<0.001) higher MV rates (p<0.001). In our multivariable regression, these associations remained with increasing odds for mortality with each stepwise increase in cardiac multimorbidity (1: OR 1.48 (1.45–1.50); 2: OR 2.13 (2.09–2.17); 3+: OR 2.43 (2.38–2.48), p<0.001, all).

**Conclusions:**

Our study is the first national examination of the impact of cardiac comorbidities on COVID outcomes. A higher number of cardiac comorbidities was associated with significantly higher rates of MV and in-hospital mortality, independent of age. Future, more granular, and longitudinal studies are needed to further examine these associations.

## Introduction

The COVID-19 pandemic has stretched healthcare resources thin and led to significant morbidity and mortality. Among patients admitted for COVID, previous institutional data has suggested that cardiac risk factors play a role in severe disease and even death.[1, 2] Possible mechanisms at play include acute myocardial infarction, lymphopenia mediated atherosclerotic plaque destabilization, and inflammatory heart dysfunction.[3–5] In addition, the impact of multimorbidity, which as been found to increase risk in other populations, is unclear.[6] Despite limited institutional or regional studies, there have been no studies utilizing national data to investigate the role of cardiac risk factors both individually and in the form of multimorbidity on outcomes of COVID hospitalizations. As a result, the aim of this study is to examine the effect of cardiac multimorbidity on healthcare utilization and outcomes among COVID hospitalizations during the first year of the pandemic.

## Methods

### Data source

The NIS (National Inpatient Sample) is a publicly available, nationally representative 20% stratified, all-payer, claims-based inpatient discharge sampling database that is collected and maintained by the Agency of Healthcare Research and Quality (AHRQ) Healthcare Cost and Utilization Project (HCUP) and represents over 35 million annual hospitalizations in the United States.[7] Since 2012, discharges in this database represent a random sample of 20% of discharges from all nonfederal U.S. hospitals, stratified by hospital, census division, ownership status, urban vs rural location, teaching status, bed size, patient diagnosis-related group, and admission month. We followed the recommendation from the AHRQ for analysis using survey data, using patient-level and hospital-level trend weights provided to obtain national estimates. In accordance with standard practices of utilizing NIS, we also acknowledge that observations are hospitalization events not people, we have not performed state level analysis, we have not limited our data analysis years to pre-2011, we have not performed physician level analysis, and have not used non-specific secondary diagnosis codes. Additionally, we have used survey specific analysis with appropriate weighting and accounted for changes during the major transition periods of the dataset.[8]

### Statistical analysis

Using the national inpatient sample (NIS), we identified all adult hospital admissions with a primary diagnosis of COVID in 2020, using International Classification of Diseases, Tenth Revision, Clinical Modification (ICD-10-CM) codes (U07.1). Coronary artery disease (CAD), diabetes mellitus (DM), heart failure (HF), peripheral vascular disease (PVD), previous stroke, and atrial fibrillation (AF) were then identified as cardiac comorbidities using ICD-10-CM codes within the secondary diagnosis records within the NIS database (COVID-19 was the primary diagnosis code) (**S1 Table**).[9] Chi-squared tests were used for categorical variable comparisons and Kruskal Wallis tests were utilized for continuous variables given the non-standard distribution of continuous variable data. Multivariable logistic regression was used to evaluate the effect of cardiac multimorbidity on mortality and mechanical ventilation (MV). Our multivariable model included age, sex, race, comorbidities, hospital location/teaching status, hospital size, hospital region, primary expected payer, and household income of patient’s ZIP code. In the regression examining individual comorbidities, each comorbidity was individually included as variables, however for the regression examining cardiac multi-morbidity (0, 1, 2, 3+ comorbidities), individual comorbidities were not included in the regression to prevent collinearity. A sensitivity analysis was performed where patients were stratified into groups by age: 18–44 years, 45–64 years, 65–79 years, and 80+ years. Statistical analyses were performed using SPSS v26 (IBM, Armonk, NY). Given multiple testing within this study, Bonferroni correction was applied to our primary analysis with statistical significance for our descriptive testing set to p<0.001. This study was exempt from IRB approval given its use of public, de-identified data.

## Results

**Fig 1:**
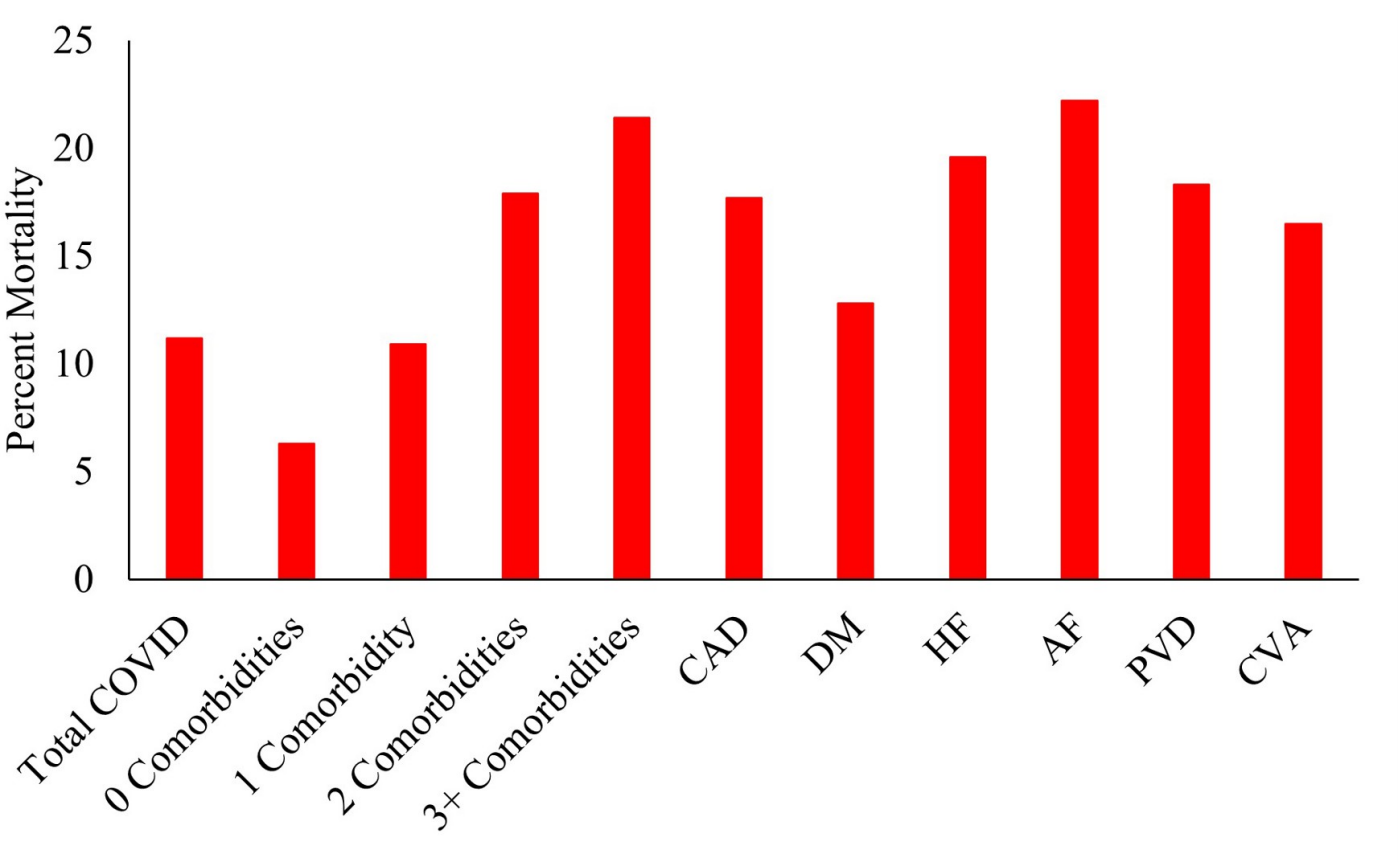
Mortality rates by cardiac comorbidity and cardiac multi-morbidity.

We identified 1,005,040 primary COVID admissions in 2020. Of these admissions, 216,545 (20.6%) had CAD, 413,195 (39.4%) had DM, 176,780 (16.8%) had HF, 159,700 (15.2%) had AF, 30735 (2.9%) had PVD, and 25,155 (2.4%) had a previous stroke. When stratified by number of comorbidities, 428390 (40.8%) had 0 comorbidities, 354960 (33.8%) had 1, 161225 (15.4%) had 2, and 105465 (10.0%) had 3+ comorbidities. COVID hospitalizations with higher cardiac multimorbidity had higher mortality rates (0: 6.3%, 1: 10.9%, 2: 17.9%, 3+: 21.4%, p<0.001) (**Fig 1**) higher MV rates (0: 6.7%, 1: 10.6%, 2: 13.8%, 3+: 14.0%, p<0.001), and lower rates of home discharge among those who survived their hospitalization (0: 77.1%, 1: %, 2: 65.6%, 3+: 59.4%, p<0.001). Those with higher cardiac multimorbidity also had longer length of stay in days (0: 5.0 [3.0–8.0], 1: 5.0 [3.0–9.0], 2: 6.0 [3.0–11.0], 3+: 6.0 [4.0–11.0], p<0.001) and higher hospital costs. (0: 9256 [5392–16669], 1: 10898 [6182–20677], 2: 12043 [6793–23617], 3+: 12873 [7197–24172], p<0.001). Associated data can be found in **Table 1**.

**Table 1:** Demographics and comorbidities.

Variables	0 Comorbidities (N = 428,390)	1 Comorbidity (N = 354,960)	2 Comorbidities (N = 161,225)	3+ Comorbidities (N = 105,465)	P-Value
**Age, years**	60.0 [47.0–72.0]	66.0 [55.0–77.0]	73.0 [64.0–82.0]	76.0 [68.0–83.0]	**<0.001**
**Female (%)**	49.2	48.4	44.0	39.9	**<0.001**
**Race (%)**					**<0.001**
White	50.6	48.5	59.4	64.8	
Black	17.0	20.1	19.3	17.8	
Hispanic	23.5	22.5	14.3	11.7	
Asian or Pacific Islander	3.4	3.4	2.9	2.4	
Native American	0.9	1.3	0.9	0.7	
Unknown	4.7	4.2	3.2	2.6	
**Comorbidities (%)**					
CAD	0.0	13.3	51.4	82.1	-
DM	0.0	65.2	63.1	75.3	-
HF	0.0	7.3	39.7	82.4	-
AF	0.0	10.4	34.2	64.3	-
PVD	0.0	2.1	6.1	12.7	-
CVA	0.0	1.6	5.5	10.0	-
**Hospital Type (%)**					**<0.001**
Rural	11.1	11.9	12.7	12.0	
Urban Non-Teaching	19.7	19.4	18.7	18.2	
Urban Teaching	69.1	68.6	68.6	69.8	
**Hospital Bed Size (%)**					**<0.001**
Small	26.3	25.5	25.1	24.4	
Medium	29.0	28.8	28.9	29.2	
Large	44.7	45.7	46.0	46.3	
**Hospital Region (%)**					**<0.001**
Northeast	18.2	16.9	17.9	17.6	
Midwest	21.8	22.3	25.6	29.1	
South	42.0	42.6	41.4	39.3	
West	18.0	18.1	15.1	14.0	
**Payer Information (%)**					**<0.001**
Medicare	38.2	52.0	72.3	80.2	
Medicaid	13.7	12.5	7.8	6.0	
Private Insurance	37.1	27.1	15.4	10.4	
Median Household Income* (%)					**<0.001**
Quartile 1 (Lowest)	32.2	36.1	35.5	34.5	
Quartile 2	27.2	27.7	28.1	28.9	
Quartile 3	22.8	21.3	21.3	21.3	
Quartile 4 (Highest)	17.9	14.9	15.1	15.3	
**Mechanical Ventilation**	6.7	10.6	13.8	14.0	**<0.001**
**Discharge Disposition (%)**					**<0.001**
** Died in Hospital**	6.3	10.9	17.9	21.4	**<0.001**
Routine	67.5	54.7	37.2	28.8	
Transfer to Short Term Hospital	3.0	3.1	2.8	2.5	
Skilled Nursing Facility*	11.4	16.3	24.5	28.6	
Home Health Care	10.7	13.9	16.7	17.9	
Against Medical Advice	1.1	1.0	0.9	0.8	
**Length of Stay, days; median**	5.0 [3.0–8.0]	5.0 [3.0–9.0]	6.0 [3.0–11.0]	6.0 [4.0–11.0]	**<0.001**
**Total Hospital Costs, 2020 USD**	9256 [5393–16670]	10898 [6183–20677]	12044 [6794–23617]	12873 [7197–24172]	**<0.001**

**Fig 2:**
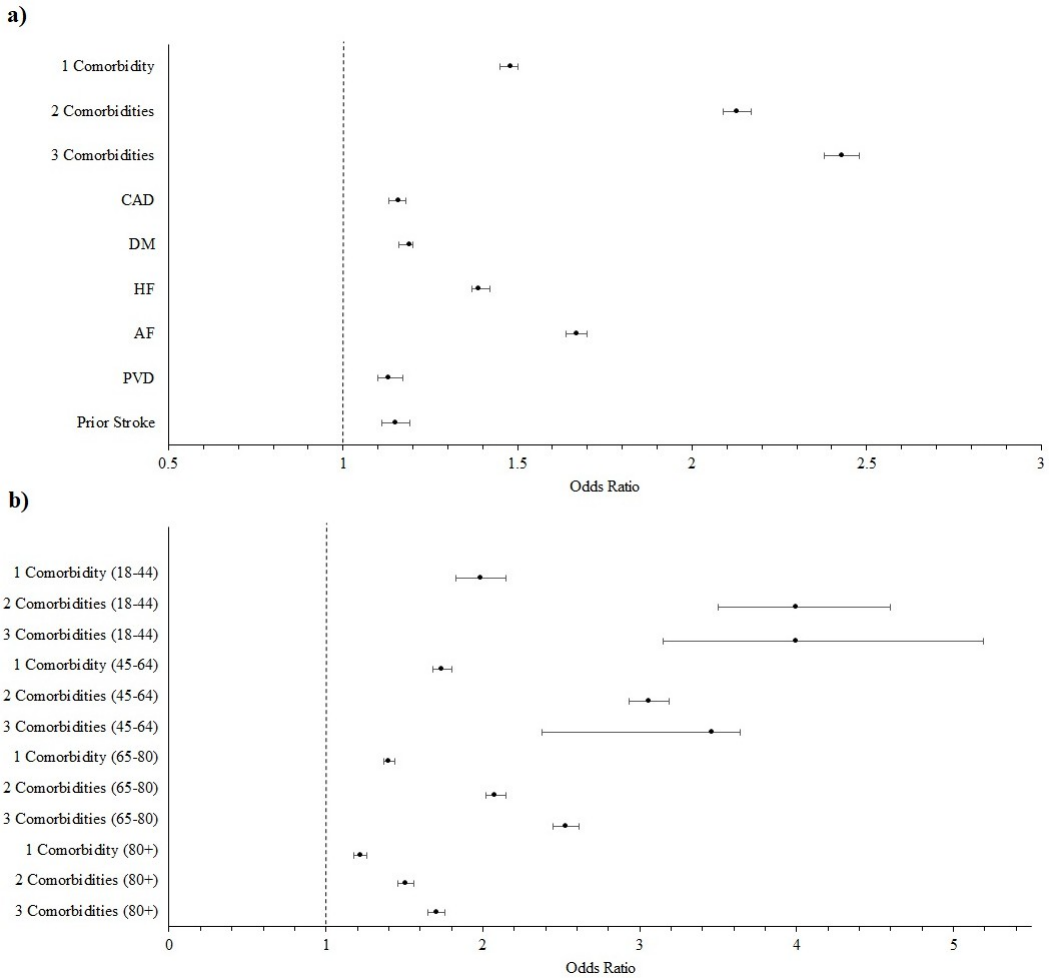
**a)** Forest Plot displaying odds of mortality by comorbidities; **b)** Forest Plot displaying odds of mortality by cardiac multi-morbidity stratified by age group.

In our multivariable regression, these associations remained with increasing odds for mortality with each stepwise increase in cardiac multimorbidity (1: OR 1.48 (1.45–1.50); 2: OR 2.13 (2.09–2.17); 3+: OR 2.43 (2.38–2.48), p<0.001, all) and with each individual comorbidity (Prior Stroke: OR 1.15 (1.11–1.19); PVD: OR 1.13 (1.10–1.17); AF: OR 1.67 (1.64–1.70); HF OR 1.39 (1.37–1.42); DM: OR 1.19 (1.17–1.20); CAD: OR 1.16 (1.14–1.18), p<0.001, all) (**Fig 2A**). Additionally, cardiac multimorbidity was associated with higher odds of MV (1: OR 1.67 (1.64–1.70); 2: OR 2.37 (2.32–2.42); 3+: OR 2.41 (2.36–2.47), p<0.001, all) and individual comorbidities (except for PVD and prior stroke) were independently associated with higher odds of MV (AF: OR 1.86 (1.83–1.90); HF OR 1.31 (1.29–1.33); DM: OR 1.37 (1.35–1.38); CAD: OR 1.10 (1.08–1.11), p<0.001, all).

We additionally conducted a sensitivity analysis in which patients were stratified into age groups. Mortality trends remained similar and can be found in **Fig 2B**.

## Discussion

This study demonstrated that cardiac comorbidities and multimorbidity were associated with significantly greater resource utilization and increased risk for mortality and MV. Interestingly, there was a stepwise increase in risk for worse outcomes with increased cardiac multimorbidity. This study also was one of the first to demonstrate the connection between increased cardiac multimorbidity and increased healthcare utilization, specifically direct cost, hospital length of stay and home discharges. In regard to the individual comorbidities, atrial fibrillation had the strongest association with worse outcomes. This echoes previous data from Denmark which demonstrated increased risk of severe COVID-19 with atrial fibrillation and heart failure.[10] It is possible that this is due the prothrombotic nature of COVID which in turn could increase the already elevated likelihood of thrombus formation in patients with concurrent atrial fibrillation.[11] More generally, this study echoes previous data which demonstrated that cardiovascular disease in COVID-19 patients was associated with worsened mortality, however our study adds a nationwide analysis of the United States to this fund of data, in addition to examining resource utilization.[12–18]

In a secondary analysis, we investigated if cardiac multimorbidity was a driver of worse outcomes independent of age. Advanced age has become established risk factor for severe illness and death due to COVID-19 with underlying immunologic mechanisms purposed.[19] In our analysis we determined that cardiac multimorbidity, independent of age has a significant effect on worse outcomes. It appears that comorbidities had a weaker association with mortality as age increased, however the significant association with mortality remained, even when only examining patients over the age of 80. As a result, it is important for physicians to take comorbidities into effect when risk stratifying patients regardless of their age. Additionally, it is important that future studies examine the effects of non-cardiac comorbidities such as end stage renal disease, liver disease, pulmonary diseases, and to examine the interplay between them and age to better understand the mortality and morbidity burden of COVID-19 on the US healthcare system.

Many of the limitations of this study stem from the NIS database itself. Given the nature of the database and ICD codes, there is a chance of misidentification or under-identification of comorbidities due to code sensitivity. Limited testing capabilities in 2020 may also have contributed to an under-identification of COVID-19 infection. The NIS does not allow for evaluation of the severity of the COVID-19 infection and does not allow for the identification of intensive care unit stays. Finally, this study is observational, and the variables acquired from the NIS are not longitudinal, limiting our ability to identify patient outcomes beyond their hospitalization.

## Conclusions

Our study is the first national examination of the impact of cardiac comorbidities on COVID outcomes, demonstrating worse outcomes across all age groups. A higher number of cardiac comorbidities was associated with significantly higher rates of MV and in-hospital mortality, independent of age. Future, more granular, and longitudinal studies are needed to further examine these associations.

## Supporting information

S1 Table ICD-10 codes for classification of diagnoses. (DOCX)
