# Supplementary material for: Brief report: Effect of cardiac multi-morbidity on COVID hospitalization outcomes
Source: PLoS One. 2024 Apr 24;19(4):e0301898. doi: 10.1371/journal.pone.0301898 (PMC11042697; doi:10.1371/journal.pone.0301898)
Supplement: S1 Table — (DOCX) [file pone.0301898.s001.docx]

**eTable 1.** ICD-10 codes for classification of diagnoses

| **Diagnoses** | **ICD-10** |
| --- | --- |
| COVID-19 | ‘U07.1’ |
| Coronary Artery Disease | 'I20', 'I200', 'I201', 'I21', 'I210', 'I2101', 'I2102', 'I2019', 'I211', 'I2111', 'I2119', 'I212', 'I2121', 'I2129', 'I213', 'I214', 'I219', 'I22', 'I220', 'I221', 'I228', 'I229', 'I23', 'I230', 'I231', 'I232', 'I233', 'I234', 'I235', 'I236', 'I237', 'I238', 'I240', 'I241', 'I248', 'I249', 'I251', 'I2510', 'I2511', 'I25110', 'I25111', 'I25118', 'I25119', 'I252', 'I253', 'I254', 'I2541', 'I2542', 'I255', 'I256', 'I257', 'I2570', 'I25700', 'I25702', 'I25708', 'I25709', 'I2571', 'I25720', 'I25711', 'I25718', 'I25719', 'I2572', 'I25720', 'I25721', 'I25728', 'I25729', 'I2573', 'I25730', 'I25731', 'I25738', 'I25739', 'I2575', 'I25750', 'I25751', 'I25758', 'I25759', 'I2576', 'I25760', 'I25761', 'I25768', 'I25769', 'I2579', 'I25790', 'I25791', 'I25798', 'I25799', 'I258', 'I2581', 'I25810', 'I25811', 'I25812', 'I2582', 'I2583', 'I2584', 'I2589', 'I259' |
| Diabetes Mellitus | 'E10', 'E100', 'E1000', 'E1001', 'E101', 'E1010', 'E1011', 'E102', 'E1021', 'E1022', 'E1029', 'E103', 'E1031', 'E10311', 'E10319', 'E1032', 'E10321', 'E103211', 'E103212', 'E103213', 'E103219', 'E10329', 'E103291', 'E103292', 'E103293', 'E103299', 'E1033', 'E10331', 'E103311', 'E103312', 'E103313', 'E103319', 'E10339', 'E103391', 'E103392', 'E103393', 'E103399', 'E1034', 'E10341', 'E103411', 'E103412', 'E103413', 'E103419', 'E10349', 'E103491', 'E103492', 'E103493', 'E103499', 'E1035', 'E10351', 'E103511', 'E103512', 'E103513', 'E103519', 'E10352', 'E103521', 'E103522', 'E103523', 'E103529', 'E10353', 'E103531', 'E103532', 'E103533', 'E103539', 'E10354', 'E103541', 'E103542', 'E103543', 'E103549', 'E10355', 'E103551', 'E103552', 'E103553', 'E103559', 'E10359', 'E103591', 'E103592', 'E103593', 'E103599', 'E1036', 'E1037', 'E1037X1', 'E1037X2', 'E1037X3', 'E1037X9', 'E1039', 'E104', 'E1040', 'E1041', 'E1042', 'E1043', 'E1044', 'E1049', 'E105', 'E1051', 'E1052', 'E1059', 'E106', 'E1061', 'E10610', 'E10618', 'E1062', 'E10620', 'E10621', 'E10622', 'E10628', 'E1063', 'E10630', 'E10638', 'E1064', 'E10641', 'E10649', 'E1065', 'E1069', 'E108', 'E109',  'E11', 'E110', 'E1100', 'E1101', 'E111', 'E1110', 'E1111', 'E112', 'E1121', 'E1122', 'E1129', 'E113', 'E1131', 'E11311', 'E11319', 'E1132', 'E11321', 'E113211', 'E113212', 'E113213', 'E113219', 'E11329', 'E113291', 'E113292', 'E113293', 'E113299', 'E1133', 'E11331', 'E113311', 'E113312', 'E113313', 'E113319', 'E11339', 'E113391', 'E113392', 'E113393', 'E113399', 'E1134', 'E11341', 'E113411', 'E113412', 'E113413', 'E113419', 'E11349', 'E113491', 'E113492', 'E113493', 'E113499', 'E1135', 'E11351', 'E113511', 'E113512', 'E113513', 'E113519', 'E11352', 'E113521', 'E113522', 'E113523', 'E113529', 'E11353', 'E113531', 'E113532', 'E113533', 'E113539', 'E11354', 'E113541', 'E113542', 'E113543', 'E113549', 'E11355', 'E113551', 'E113552', 'E113553', 'E113559', 'E11359', 'E113591', 'E113592', 'E113593', 'E113599', 'E1136', 'E1137', 'E1137X1', 'E1137X2', 'E1137X3', 'E1137X9', 'E1139', 'E114', 'E1140', 'E1141', 'E1142', 'E1143', 'E1144', 'E1149', 'E115', 'E1151', 'E1152', 'E1159', 'E116', 'E1161', 'E11610', 'E11618', 'E1162', 'E11620', 'E11621', 'E11622', 'E11628', 'E1163', 'E11630', 'E11638', 'E1164', 'E11641', 'E11649', 'E1165', 'E1169', 'E118', 'E119',  'E12', 'E120', 'E1200', 'E1201', 'E121', 'E1210', 'E1211', 'E122', 'E1221', 'E1222', 'E1229', 'E123', 'E1231', 'E12311', 'E12319', 'E1232', 'E12321', 'E123211', 'E123212', 'E123213', 'E123219', 'E12329', 'E123291', 'E123292', 'E123293', 'E123299', 'E1233', 'E12331', 'E123311', 'E123312', 'E123313', 'E123319', 'E12339', 'E123391', 'E123392', 'E123393', 'E123399', 'E1234', 'E12341', 'E123411', 'E123412', 'E123413', 'E123419', 'E12349', 'E123491', 'E123492', 'E123493', 'E123499', 'E1235', 'E12351', 'E123511', 'E123512', 'E123513', 'E123519', 'E12352', 'E123521', 'E123522', 'E123523', 'E123529', 'E12353', 'E123531', 'E123532', 'E123533', 'E123539', 'E12354', 'E123541', 'E123542', 'E123543', 'E123549', 'E12355', 'E123551', 'E123552', 'E123553', 'E123559', 'E12359', 'E123591', 'E123592', 'E123593', 'E123599', 'E1236', 'E1237', 'E1237X1', 'E1237X2', 'E1237X3', 'E1237X9', 'E1239', 'E124', 'E1240', 'E1241', 'E1242', 'E1243', 'E1244', 'E1249', 'E125', 'E1251', 'E1252', 'E1259', 'E126', 'E1261', 'E12610', 'E12618', 'E1262', 'E12620', 'E12621', 'E12622', 'E12628', 'E1263', 'E12630', 'E12638', 'E1264', 'E12641', 'E12649', 'E1265', 'E1269', 'E128', 'E129',  'E13', 'E130', 'E1300', 'E1301', 'E131', 'E1310', 'E1311', 'E132', 'E1321', 'E1322', 'E1329', 'E133', 'E1331', 'E13311', 'E13319', 'E1332', 'E13321', 'E133211', 'E133212', 'E133213', 'E133219', 'E13329', 'E133291', 'E133292', 'E133293', 'E133299', 'E1333', 'E13331', 'E133311', 'E133312', 'E133313', 'E133319', 'E13339', 'E133391', 'E133392', 'E133393', 'E133399', 'E1334', 'E13341', 'E133411', 'E133412', 'E133413', 'E133419', 'E13349', 'E133491', 'E133492', 'E133493', 'E133499', 'E1335', 'E13351', 'E133511', 'E133512', 'E133513', 'E133519', 'E13352', 'E133521', 'E133522', 'E133523', 'E133529', 'E13353', 'E133531', 'E133532', 'E133533', 'E133539', 'E13354', 'E133541', 'E133542', 'E133543', 'E133549', 'E13355', 'E133551', 'E133552', 'E133553', 'E133559', 'E13359', 'E133591', 'E133592', 'E133593', 'E133599', 'E1336', 'E1337', 'E1337X1', 'E1337X2', 'E1337X3', 'E1337X9', 'E1339', 'E134', 'E1340', 'E1341', 'E1342', 'E1343', 'E1344', 'E1349', 'E135', 'E1351', 'E1352', 'E1359', 'E136', 'E1361', 'E13610', 'E13618', 'E1362', 'E13620', 'E13621', 'E13622', 'E13628', 'E1363', 'E13630', 'E13638', 'E1364', 'E13641', 'E13649', 'E1365', 'E1369', 'E138', 'E139',  'E14', 'E140', 'E1400', 'E1401', 'E141', 'E1410', 'E1411', 'E142', 'E1421', 'E1422', 'E1429', 'E143', 'E1431', 'E14311', 'E14319', 'E1432', 'E14321', 'E143211', 'E143212', 'E143213', 'E143219', 'E14329', 'E143291', 'E143292', 'E143293', 'E143299', 'E1433', 'E14331', 'E143311', 'E143312', 'E143313', 'E143319', 'E14339', 'E143391', 'E143392', 'E143393', 'E143399', 'E1434', 'E14341', 'E143411', 'E143412', 'E143413', 'E143419', 'E14349', 'E143491', 'E143492', 'E143493', 'E143499', 'E1435', 'E14351', 'E143511', 'E143512', 'E143513', 'E143519', 'E14352', 'E143521', 'E143522', 'E143523', 'E143529', 'E14353', 'E143531', 'E143532', 'E143533', 'E143539', 'E14354', 'E143541', 'E143542', 'E143543', 'E143549', 'E14355', 'E143551', 'E143552', 'E143553', 'E143559', 'E14359', 'E143591', 'E143592', 'E143593', 'E143599', 'E1436', 'E1437', 'E1437X1', 'E1437X2', 'E1437X3', 'E1437X9', 'E1439', 'E144', 'E1440', 'E1441', 'E1442', 'E1443', 'E1444', 'E1449', 'E145', 'E1451', 'E1452', 'E1459', 'E146', 'E1461', 'E14610', 'E14618', 'E1462', 'E14620', 'E14621', 'E14622', 'E14628', 'E1463', 'E14630', 'E14638', 'E1464', 'E14641', 'E14649', 'E1465', 'E1469', 'E148', 'E149' |
| Heart Failure | '150', 'I500', 'I501', 'I502', 'I5020', 'I5021', 'I5022', 'I5023', 'I503', 'I5030', 'I5031', 'I5032', 'I5033', 'I504', 'I5040', 'I5041', 'I5042', 'I5043', 'I508', 'I5081', 'I50810', 'I50811', 'I50812', 'I50813', 'I50814', 'I5082', 'I5083', 'I5084', 'I5089', 'I509', 'I110', 'I130', 'I132', 'I099', 'I255', 'I420', 'I421', 'I422', 'I425', 'I426', 'I427', 'I428', 'I429', 'I43' |
| Peripheral Vascular Disease | 'I70', 'I700', 'I701', 'I702', 'I708', 'I709', 'I71', 'I710', 'I711', 'I712', 'I713', 'I714', 'I715', 'I716', 'I718', 'I719', 'I731', 'I738', 'I739', 'I771', 'I790', 'I792', 'K551', 'K558', 'K559', 'Z958', 'Z959' |
| Previous Stroke | 'I60', 'I600', 'I6000', 'I6001', 'I6002', 'I601', 'I6010', 'I6011', 'I6012', 'I602', 'I603', 'I6030', 'I6031', 'I6032', 'I604', 'I605', 'I6050', 'I6051', 'I6052', 'I606', 'I607', 'I608', 'I609', 'I61', 'I610', 'I611', 'I612', 'I613', 'I614', 'I615', 'I616', 'I618', 'I619', 'I63', 'I630', 'I6300', 'I6301', 'I63011', 'I63012', 'I63013', 'I63019', 'I6302', 'I6303', 'I63031', 'I63032', 'I63033', 'I63039','I6309', 'I631', 'I6310', 'I6311', 'I63111', 'I63112', 'I63113', 'I63119', 'I632', 'I6320', 'I6321', 'I63211', 'I63212', 'I63213', 'I63219', 'I6322', 'I6323', 'I63231', 'I63232', 'I63233', 'I63239', 'I6329', 'I633', 'I6330', 'I6331', 'I63311', 'I63312', 'I63313', 'I63319', 'I6332', 'I63321', 'I63322', 'I63323', 'I63329', 'I6333', 'I63331', 'I63332', 'I63333', 'I63339', 'I634', 'I6340', 'I6341', 'I63411', 'I63412', 'I63413', 'I63419', 'I6342', 'I63421', 'I63422', 'I63423', 'I63429', 'I6343', 'I63431', 'I63432', 'I63433', 'I63439', 'I6344', 'I63441', 'I63442', 'I63443', 'I63449', 'I6349', 'I635', 'I6350', 'I6351', 'I63511', 'I63512', 'I63513', 'I63519', 'I6352', 'I63521', 'I63522', 'I63523', 'I63529', 'I6353', 'I63531', 'I63532', 'I63533', 'I63539', 'I6354', 'I63541', 'I63542', 'I63543', 'I63549', 'I6359', 'I636', 'I638', 'I6381', 'I6389', 'I639' |
| Atrial Fibrillation | '4273', '42730', '42731', '42732' |
